# Local Fiscal Pressure and Public Health: Evidence from China

**DOI:** 10.3390/ijerph20065126

**Published:** 2023-03-14

**Authors:** Xu Zhang, Tianchu Feng, Chengjun Wang, Chaozhu Li

**Affiliations:** 1 School of Economics and Management, Zhejiang Agriculture and Forestry University, Hangzhou 311300, China; 2 Jiyang College, Zhejiang Agriculture and Forestry University, Zhuji 311800, China; 3 China Institute for Rural Studies, Tsinghua University, Beijing 100084, China

**Keywords:** local fiscal pressure, public health, public health expenditure, industrial updating, environmental pollution

## Abstract

Under the dual challenges of global downward economic pressure and the COVID-19 pandemic, studying the impact of local government fiscal pressure on public health is a meaningful endeavor. First, this paper analyzes the impact of local government fiscal pressure on public health and clarifies its impact mechanisms. Second, by utilizing panel data of 31 Chinese provinces from 2000 to 2020, two-way fixed-effects and mediating-effects models are developed to identify the effects and impact mechanisms of local government fiscal pressure on public health. The results show that local government fiscal pressure can be detrimental to public health through three main mechanisms: reducing public health fiscal expenditures, hindering industrial structure upgrading, and exacerbating environmental pollution. Heterogeneity analysis finds that the negative effects of local government fiscal pressure on public health mainly exist in Central and Western China. Accordingly, three policy implications are proposed: optimizing the fiscal system, accelerating industrial upgrading, and improving the appraisal system of local officers.

## 1. Introduction

Public health has long been a crucial topic, and governments have been attempting to improve public health in their respective countries. For example, since 2015, global domestic general government health expenditure (GGHE-D) as percentage of general government expenditure (GGE) has been above 10% and has shown a continuous increase. In the Americas, GGHE-D as percentage of GGE was as high as 14.3% in 2020 [1]. Similarly, the Chinese government is actively improving public health, as seen in the 11.3% increase in budgetary allocations that the Chinese Health Council received from the state treasury in 2020 relative to 2019. By the end of November 2020, the Chinese government had spent over CNY 400 billion (USD 61.73 billion) in financial resources to address the COVID-19 pandemic. In 2019, the Chinese central government requested an improvement in public health. It proposed an insistence on placing the protection of people and human life first, moving people’s health to the forefront of its development strategy, and formulating a major national strategy for a “healthy China”. However, in early 2020, the COVID-19 pandemic began, and currently poses a severe threat to public health worldwide [2]. This situation reflects the continued shortcomings in the relative level of public health in various countries when faced with emergencies. Accordingly, studying public health issues is very relevant undertaking.

Government finances are the cornerstone of funding for public health [3]. However, since the implementation of the tax-sharing reform in China in 1994, the fiscal constraints on local governments have become severe, and local government fiscal pressure (LFP) has become increasingly serious. In 2003, the central and local enterprise income tax ratio was adjusted to 60:40. Since the value-added tax reform in 2016, the central and local value-added tax ratio has been 50:50. The tax-sharing reform has enabled the central government to receive most of the tax revenues, and local governments have taken on many expenditure responsibilities regarding public goods and economic development while receiving limited tax revenue. The long-term imbalance between revenues and expenditures has led to an increasing fiscal gap for local governments and increasingly severe LFP [4]. This phenomenon has received increasing academic attention, and many scholars have analyzed the impact of LFP on economic growth, pollution emissions, and technological innovation from the perspective of government behavioral response [5,6]. Moreover, a few studies have focused on the relationship between vertical fiscal imbalances and fiscal health expenditure efficiency, along with the impact of fiscal decentralization on public health [7,8]. However, attention has been seldom given to the impact of LFP on public health, and its impact effects and mechanisms are not clarified. Accordingly, this paper theoretically analyzes the effects of LFP on public health and its mechanisms. In addition, by utilizing panel data of 31 Chinese provinces from 2000 to 2020, two-way fixed effects and mediating-effects models are developed to empirically analyze the impact effects and impact mechanisms of LFP on public health. These models are beneficial in analyzing and understanding the impact of LFP on public health more clearly. We find that local government fiscal pressure can be detrimental to public health through three main mechanisms: reducing public health fiscal expenditures, hindering industrial structure upgrading, and exacerbating environmental pollution. The results are still robust after a series of robustness checks. Moreover, heterogeneity analysis finds that the negative effects of local government fiscal pressure on public health mainly exist in Central and Western China. Based on these results, we propose three policy implications.

Compared with previous studies, the possible marginal contribution of this paper is mainly reflected in the following aspects. First, there is a lack of studies focusing on the impact of local fiscal pressure and public health. Furthermore, this study reveals the effects of local fiscal pressure on governmental behavior change in terms of public health. Second, some studies focus on the effects of fiscal decentralization and vertical fiscal imbalance on public health [7,8], but they focus only on the mechanisms of the effects of curtailing public health expenditures and ignore the effects of other factors on public health due to changes in government behavior. Through theoretical and empirical analyses, this study elucidates multiple mechanisms by which LFP affects public health in addition to reducing health expenditures. Third, a two-way fixed effects model and the two-stage least squares (2SLS) model are used to solve the endogeneity problem and ensure the robustness of the study results. Lastly, we use the basic results as a basis for conducting a regional heterogeneity analysis. The results of this study will expand our understanding of the consequences of LFP and provide policy implications for the government to improve public health in the face of various challenges, such as the COVID-19 pandemic.

The remainder of this paper is structured as follows. Section 2 summarizes the relevant literature and constructs the theoretical mechanism of the LFP effect on public health. Section 3 introduces the analysis strategy and data explanation. Section 4 presents the empirical results. Lastly, Section 5 provides the conclusions and policy implications.

## 2. Literature Review and Hypothesis Development

### 2.1. Literature Review

The consequences of LFP are of immense interest to the academic community. First, numerous studies have analyzed the impact of LFP on economic growth. Previous studies have argued that moderate LFP would promote economic growth because the government would tend to support local tax-focused enterprises and local high-tax industries (e.g., property, construction, and industry), thereby driving economic growth [9]. However, unbalanced industrial development caused by excessive fiscal pressure can lead to unsustainable economic development [10,11].

Some scholars have also focused on the impact of LFP on local government behavior. Numerous international studies have demonstrated that LFP may distort the structure of public expenditure and cut public service expenditures [12,13,14,15,16]. For Chinese governments, Fu and Zhang (2007) argued that, as China’s LFP intensifies, most local governments focus on effective capital construction investment while disregarding investments in education, culture, and health [17]. Li and Du (2017) argued that the presence of LFP generates more short-sighted behavior in local governments in China [18]. To obtain rich and stable tax sources, numerous factor resources have been flowing to the industries that can generate GDP rapidly [19]. The industrial structure will also be adjusted to favor the secondary industry, with extensive physical and human capital being concentrated there [20].

Faced with fiscal pressures, governments often choose to implement austerity policies in addition to restructuring their spending. Several articles have explored the impact of austerity on public health, but no uniform conclusion has been reached. Generally, studies have concluded that austerity policies have a negative impact on public health through economic factors with a healthcare effect, such as increased unemployment, homelessness, and poverty, along with reductions in health coverage and restricted access to care [21,22,23]. However, some studies have suggested the opposite. Rajmil and Fernández (2019) found in Europe that there is a negative effect on mortality in the countries that apply a higher level of austerity, based on Eurosta data [24]. Perotti (2021) evaluated austerity in Greece by means of descriptive statistics and comparisons, concluding that a rise in self-perceived health occurred when austerity measures were imposed. In addition, the rate of the decline in the standardized mortality rate reduced during the crisis [25].

Studies have also demonstrated the impact of LFP on government public sector efficiency. Christl et al. (2020) used data from 23 European countries for the 1995–2015 period to construct public sector efficiency indicators. They found that LFP is associated with low public sector efficiency [26]. Other studies have analyzed the impact of LFP on separate sector efficiency, such as energy and green environmental efficiencies. Feng et al. (2023) used Chinese provincial panel data from 2001 to 2017 in examining the relationship between vertical fiscal imbalances and energy efficiency through a mediation model [27]. Their results showed that vertical fiscal imbalances substantially suppress energy efficiency by reducing government spending on science and technology. LFP stimulates local governments to emphasize productive expenditures considerably, reduce environmental governance expenditures, and reduce green development efficiency [28,29]. Hui et al. (2022) analyzed the impact of LFP on air quality in China and found that LFP has a significant negative impact on air quality, particularly in areas with high levels of industrialization [30]. Qin (2022) investigated the relationship between LFP and water pollution in China and found that LFP has a significant negative impact on water quality, particularly in coastal regions [31]. 

Some studies have analyzed the impact of vertical fiscal imbalances on fiscal health expenditure efficiency. Zhang and Wang (2023) argued that vertical fiscal imbalances may lead to a skewing of healthcare expenditure inputs toward the more profitable superior hospitals, thereby exacerbating the resource imbalance among different healthcare institutions and reducing healthcare expenditure efficiency [7]. Nevertheless, vertical fiscal imbalances may also improve healthcare efficiency through the corrective effect of central transfers. The paper also verifies that vertical fiscal imbalances have a positive effect on healthcare efficiency, but it gradually turns from positive to negative along with improvements in fiscal health expenditure efficiency. However, the paper does not analyze the effect of LFP on public health and the mechanism of the effect. Other studies related to this paper investigate the impact of fiscal decentralization on public health. However, scientifically measuring fiscal decentralization is a complicated task, as different measurements may give rise to different results and conclusions [32,33,34,35]. Moreover, fiscal decentralization is determined by the central government to a large extent [36,37]. Thus, the model results may not be robust due to less variation among the provinces. Therefore, discussing the relationship between government behavior and public health from the perspective of LFP may have more applicability and practical significance for China.

In summary, previous studies have analyzed the impact of LFP with regard to various aspects, such as economic growth, local government behavior, and public sector efficiency. However, there is a lack of studies focusing on the impact of local fiscal pressure and public health. The most relevant articles to this paper are those by Xu and Lin (2023) and Zhang and Wang (2023), which discuss the impact of fiscal decentralization on public health and the impact of vertical fiscal imbalances on the efficiency of healthcare spending, respectively [7,8]. However, these articles focus only on the effects of public health expenditures and ignore other possible mechanisms of influence. In addition, some studies have discussed the effects on public health of government choices to cut spending in the face of fiscal pressures but have not reached uniform conclusions. Therefore, to study the impact of local governments on public health, we need to analyze not only the direct effects of fiscal austerity on public health, but also other possible mechanisms of influence. In this paper, we focus on the direct impact mechanism of fiscal pressure on public health, as well as analyzing the indirect impact. Using panel data, we build an econometric model to exclude the impact of confounding factors on the relationship between LFP and public health and clarify multiple impact mechanisms. 

This study aims to fill the gap in the literature by examining the impact of LFP on public health and the underlying mechanisms. The results of this research will expand our understanding of the consequences of LFP and provide policy implications for the government to improve public health in the face of various challenges, such as the COVID-19 pandemic.

### 2.2. Theoretical Background

We focus on the behavioral responses and consequences of government in the face of fiscal pressures. The earliest theory on this topic is the cutback management theory [38], which argues that governments respond to fiscal pressures in a gradual manner, trying to raise taxes when the pressure is low, and cutting public services as the pressure increases. In the 21st century, under the impact of the financial crisis and the COVID-19 pandemic, some studies have revisited the cutback management theory. The research focuses on an analysis of the reflection between different governments and produces two new theories, “austerity urbanism” and “pragmatic municipalism” [39]. Austerity urbanism argues that governments, when faced with fiscal pressures, will aggressively curtail public services [40]. However, pragmatic municipalism argues that austerity urbanism exists mainly in European countries with highly centralized finances and in a very few U.S. cities, while other U.S. cities with fiscal autonomy respond to the same pressure with multiple balanced policies [41].

The promotion tournament theory is indispensable in analyzing changes in the behavior of the Chinese government [20]. The promotion tournament theory suggests that appraisal of the central government’s economic performance is key to influencing local government behavior, and that local officials will invest financial resources in areas of high economic return in order to compete with other officials and achieve their personal promotion goals. This behavior is more common in the face of fiscal pressure.

Next, we attempt to use the cutback management theory and the promotion tournament theory to analyze the behavioral responses of government to fiscal pressures and the impact on public health.

### 2.3. Hypothesis Development

The tax-sharing reform in China has exacerbated LFP on provincial governments. The main aim of the tax-sharing reform implemented by the Chinese central government in 1994 was to “shift the fiscal power upward and the service power downward.” The central government collected large amounts of tax revenue without reducing the expenditure burden on local governments [7]. The “downward pressure of authority” has resulted in provincial public health expenditures and other livelihood expenditures being overly dependent on the level of economic development, financial resources, and the extent of local government health expenditure at the provincial level. Moreover, different from the federal state’s simultaneous economic and political decentralization, the Chinese fiscal system lacks an effective and standardized transfer mechanism between the central government and the provincial local governments. LFP on provincial governments is exacerbated by the lack of an effective and regulated transfer system [42].

First, LFP has a direct impact on public health. To alleviate financial pressure, governments will naturally reduce expenditures in various areas, including public health. Additionally, at present, the promotion of local officials in China still depends on the central government [43,44]. China’s administrative governance model of promotion tournaments has led to a significant correlation between the promotion of government officials and growth in local GDP [19]. In the face of fiscal constraints, government officials often adjust the structure of government spending to ensure GDP growth to gain promotional opportunities. Therefore, provincial governments increase spending on economic goods and crowd out spending on public goods, including public health [17]. The lack of public health expenditure means that medical resources in public healthcare institutions become scarcer and the cost of public access becomes higher, both of which are detrimental to the public health of the population [45,46,47]. Therefore, we propose the first two hypotheses:

**Hypothesis** **1.** *Ceteris paribus, LFP is detrimental to public health.*

**Hypothesis** **2.** *Ceteris paribus, LFP is detrimental to public health by reducing public health expenditure.*

**Figure 1:**
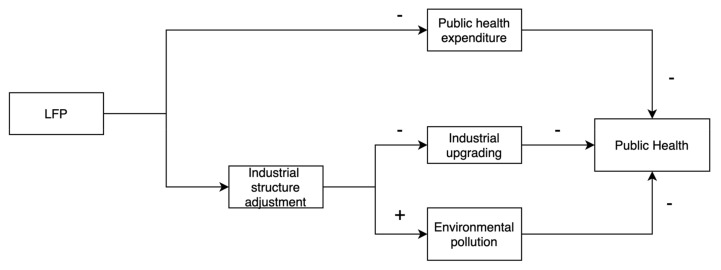
Mechanism flowchart of the impact of LFP on public health.

In addition, there are two other mechanisms. To obtain high GDP growth, governments facing LFP will adjust the industrial structure in favor of the secondary sector with high economic returns, as well as adjusting the public expenditure structure [48]. A change in industrial structure generates a significant demand for human capital, and part of the labor force shifts from primary and tertiary industries to secondary industries. Owing to the characteristics of work, the physical health and mental health of blue-collar workers are more likely to be affected by factors such as noise, heat, dust, and fumes compared with workers in other industries [49,50,51,52]. In addition, the high economic returns of the secondary sector are accompanied by high environmental pollution, with some studies proving that LFP could increase environmental pollution and relax environmental regulation [53,54,55]. Moreover, extensive research has demonstrated the health hazards resulting from environmental pollution. Kelly and Fussell (2015) found a substantial increase in findings that particulate matter (PM) air pollution exerts a significant impact on established health endpoints and is associated with numerous disease outcomes [56]. Wong et al. (2008) found that associations have been detected between most pollutants and most health outcomes under study (i.e., all-natural-cause, cardiovascular, and respiratory mortality) [57]. In addition, residents of Asian cities are more impacted by air pollution than those in Western industrial countries. Hence, this paper proposes the following hypotheses, and presents a mechanism flowchart (Figure 1):

**Hypothesis** **3.** *Ceteris paribus, LFP is detrimental to public health by hindering industrial structure upgrading.*

**Hypothesis** **4.** *Ceteris paribus, LFP is detrimental to public health by exacerbating environmental pollution.*

## 3. Method and Data

### 3.1. Empirical Models

We chose to use a two-way fixed effects model for analysis to eliminate the problem of model endogeneity caused by possible omitted variables arising from individual inherent differences that do not vary over time and year differences that vary over time. The specific model settings are as follows:

(1) $$Publichealth_{it}=\alpha +\beta_{1}lnLFP_{it}+\beta X_{it}+\mu_{i}+\gamma_{t}+\varepsilon_{it},$$

where *Publichealth_it_* is the dependent variable representing the public health of province *i* in year *t*, *LFP_it_* denotes the core independent variable local fiscal pressure, *X_it_* refers to the control variable that may affect public health, *µ_i_* represents the fixed effect of province, *λ_t_* depicts the fixed effect of year, and *ε_it_* is the random error term.

To test the effect mechanism in the hypothesis, we built a mediating effect model referring to the approach of Baron and Kenny (1986), and developed Equations (2) and (3) on the basis of Equation (1) to form a set of formulas [58]:

(2) $$Mediator_{it}=\bar{\alpha }+\bar{\beta_{1}}lnLFP_{it}+\bar{\beta }X_{it}+\mu_{i}+\gamma_{t}+\bar{\varepsilon_{it}},$$

(3) $$Publichealth_{it}=\tilde{\alpha }+\tilde{\beta_{1}}lnLFP_{it}+\tilde{\beta_{2}}Mediator_{it}+\tilde{\beta }X_{it}+\mu_{i}+\gamma_{t}+\tilde{\varepsilon_{it}},$$

where *Mediator_it_* represents the mediator variables, and the other variables are defined as in Equation (1). We are primarily interested in the significance of the regression coefficients $\beta_{1}$, $\bar{\beta_{1}}$, $\tilde{\beta_{1}}$, and $\tilde{\beta_{2}}$ in Equations (1)–(3). If they are significant and $\left|\tilde{\beta_{1}}\right|\;$ < $\left|\beta_{1}\right|$, different mediating effects of LFP on public health can be determined.

Although we add control variables and two-way fixed effects to the model to reduce the problem of omitted variables, the basic model may still have an endogeneity issue from inverse causality. When the level of local public health becomes low, the government will increase investment in public health, which intensifies LFP to a certain extent. We choose instrumental variables and establish two-stage least squares to alleviate this problem. First, we use an ordinary least squares method to estimate the impact of instrumental variables on LFP:

(4) $$\hat{lnLFP_{it}}=\theta_{1}+\theta_{2}IV_{it}+\theta_{3}X_{it}+\mu_{i}+\gamma_{t}+\mu_{it}$$

Second, we use the other model to estimate the effect of the fitting values of LFP obtained in the first stage on public health:

(5) $$Publichealth_{it}=\varphi_{1}+\varphi_{2}\hat{lnLFP_{it}}+\varphi_{3}X_{it}+\mu_{i}+\gamma_{t}+\mu_{it}$$

### 3.2. Variable Definitions

#### 3.2.1. Core Independent Variable

Fiscal pressure on local governments is caused by fiscal deficits resulting from a persistent imbalance between fiscal revenues and fiscal expenditures. In the fiscal decentralization system, the fiscal gap resulting from the imbalance between local government revenues and expenditures, or fiscal imbalance leads to local government fiscal stress [59]. Therefore, we adopt the method of Bai et al. (2018) to calculate the fiscal pressure [60]:

$$LFP_{it}=\frac{fiscalexpenditure_{it}-fiscalrevenue_{it}}{fiscalrevenue_{it}}.$$

#### 3.2.2. Dependent Variable

**Figure 2:**
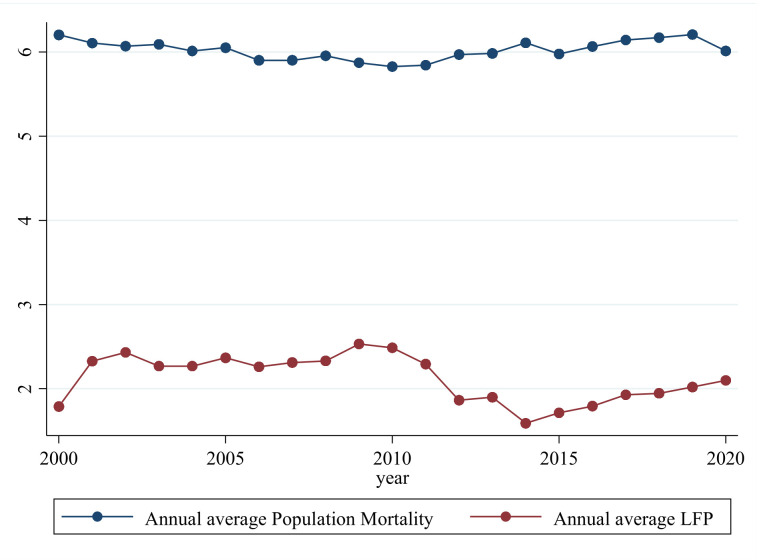
Annual average trend between LFP and population mortality. (Source: *China Statistical Yearbook* (2001–2021)).

The dependent variable for public health is a multi-dimensional macro concept. Some studies have used life expectancy per capita, infant mortality, or maternal mortality to measure public health, but the most frequently used indicator in scientific research is population mortality. Mortality is an immediate, objective indicator of the health status of the population, and perhaps the most easily measurable [25]. The higher the population mortality, the lower the public health level [8,61,62]. Therefore, we measured public health using mortality per 1000 population (*PH*). Figure 2 shows the trends in the dependent and independent variables, and as can be observed, there is essentially a correlation between LFP and population mortality.

**Figure 3:**
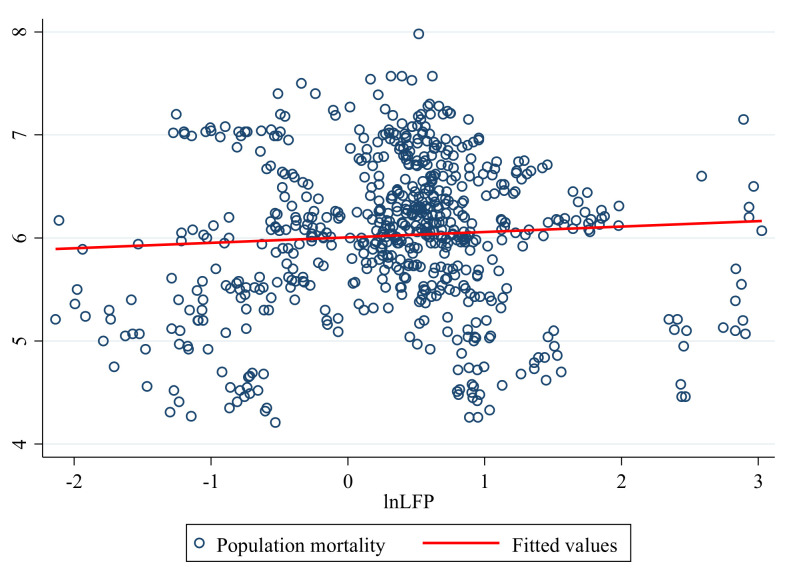
Scatter plot and fitted line between LFP and population mortality. (Source: *China Statistical Yearbook* (2001–2021)).

Furthermore, to show the correlation between LFP and mortality more clearly, we drew a scatter plot and a fit line for the sample. Figure 3 shows the correlation between LFP and population mortality, with the fitted line indicating a preliminary positive relationship between LFP and population mortality. However, the causal relationship between LFP and public health needs to be verified further.

#### 3.2.3. Mediator Variables

In the hypothesis section, we analyzed three possible mechanisms through which LFP affects public health: reducing public health expenditure, hindering the share of industrial upgrading, and increasing environmental pollution. Therefore, we selected three mediator variables to characterize public health expenditure, industry upgrading, and environmental pollution. First, we chose to characterize public health expenditure as public health spending per capital (*SPC*). Compared with using real health expenditures directly, the variable can not only remove the differences in the economic level base of each province more efficiently, but also account for variation in population size. Second, we used the ratio of employment in the secondary sector to employment in other industries to represent industry upgrading (*ESI*), which can effectively determine the transfer of employees between different secondary sectors and other industries. Lastly, we selected per 100 capita provincial annual particulate matter (PM) 2.5 to characterize environmental pollutants (*PM*). We chose PM2.5 because it has a greater correlation with public health relative to other pollutants. Research has indicated that PM with an aerodynamic diameter of below 10 µm has a greater impact on human health. One group of identified PM (i.e., PM2.5) has a small diameter but a large surface area, and may be able to carry various toxic substances that pass through the filtration of the nose hair, reaching the end of the respiratory tract via airflow and accumulating there by diffusion, damaging other parts of the body through air exchange in the lungs [63,64]. 

#### 3.2.4. Control Variables

We included control variables in the model to reduce the bias of other factors on the estimation results. First, we chose to control provincial real GDP per capita (in CNY 1000 (*GDPPC*)), and used it to control the impact of economic development on public health. Second, we controlled for provincial industrial structure (*INDS*), measured as the ratio of the value-added of secondary industries to the value-added of tertiary industries. Third, we controlled for the resident population (*RP*), which was in 10,000 people. Lastly, we controlled for urbanization level in each locality (*URBAN*), expressed using the ratio of urban population to residential population.

#### 3.2.5. Data Sources

The research sample in this paper covers China’s 31 provinces (excluding SAR) from 2000 to 2020. All data (except the PM2.5 data) are from the *China Statistical Yearbook* and the *China Finance Yearbook*. PM2.5 data were derived from the Atmospheric Composition Analysis Group [65]. Table 1 shows the descriptive statistics of the variables.

## 4. Empirical Results and Discussion

### 4.1. Basic Empirical Results Analysis

Table 2 shows the results of the basic model. First, referring to Juodis and Reese (2022), we utilized the weighted CD test statistic to test the cross-section dependence (CSD) of our sample. To reduce the dependence on the random Rademacher weights, we conducted 100 repetitions of the weighted CD test [66]. The results of $\bar{\mathrm{CDw}}$ show that the *p*-value of the weighted CD test statistic is 0.313, which means the hypothesis of weak cross-section dependence cannot be significantly rejected. This indicates that our sample has no strong cross-section dependence.

Columns 1 and 2 include the province- and year-fixed effects. Column 1 shows the model estimation results without the inclusion of the control variables. Moreover, the results show that LFP has a positive correlation with mortality and is significant at the 1% level. After adding the control variables, the results in column 2 show that LFP has a significant positive effect on population mortality. Moreover, the estimation results are still significant at the 1% level, which is a preliminary indication that the estimation results are robust. The coefficient of *lnLFP* demonstrates that when LFP increases by 1%, the population mortality rate increases by 0.004933 units; that is, when the value of LFP increases by 1.4 on average, population mortality increases by 0.4933 on average per 10 thousand people. This outcome indicates that the higher the LFP, the higher the mortality rate, and the greater the hazard to public health, holding other factors constant. Hence, the regression results validate Hypothesis 1. A study relevant to this paper is one by Xu and Lin (2022), who argue that fiscal decentralization improves public health. Our results are the opposite [8], perhaps because they only consider the mechanisms of public health spending, and ignore the impact of industrial upgrading and environmental pollution on public health.

First, for the control variables, the results in column 2 show that the higher the GDP per capita (*GDPPC*), the lower the mortality. The better the local economic development, the better it meets the population’s demand for public resources, which is consistent with the relationship between economic growth and mortality [67]. Second, for the industrial structure variable (*INDS*), the larger the ratio of value added in the secondary sector to value added in the tertiary sector, the higher the population mortality rate, which is also consistent with our theoretical expectations. Third, the larger the total local population (*POP*), the higher the mortality rate. This result may be caused by the fact that, for limited healthcare resources, the larger the population, the scarcer the resources, which is also detrimental to public health. Lastly, for the urbanization variable (*URBAN*), the provincial urbanization rate is negatively correlated with the population mortality rate. This may be due to the large urban–rural gap in China, where cities have better healthcare and transportation resources than villages, which is more favorable to public health. This result is consistent with the findings of existing studies on urbanization and mortality [68].

### 4.2. Robustness Checks

To ensure the robustness of the basic results, we used the following methods for robustness tests. The estimation results are shown in Table 3. First, in column 1, we used provincial cluster robust standard errors instead of heteroskedasticity robust standard errors for the robustness tests. Cluster robust standard errors relax the assumption of homoskedasticity across individuals, so that each province has samples in different periods in one cluster. Observations within the same cluster are correlated with each other, while observations between different clusters are not. This will reduce the model estimation efficiency, but is more realistic [69]. The estimation results in the first column show that, when using the provincial cluster robust standard errors, the standard errors of the core explanatory variables are lower than the basic results, but still positively significant at the 1% level. Second, even though we used the province- and year-fixed effects to remove the inherent differences between provinces and the inherent differences between years, to mitigate the omitted variable bias, the basic model may still have another endogeneity problem affecting the model robustness. Therefore, in column 2, we used regressions with one-period lags of the LFP variable to mitigate the model’s endogeneity problem. The regression results still show that LFP is positively correlated with mortality at the 1% level. Moreover, we opted to use a 2SLS approach for estimation, choosing the average value of LFP in other provinces in the same year (except province *i*) as the instrumental variable for the regression [70]. Column 3 presents the regression results of 2SLS and the first-stage K-P Wald F statistic, showing that the K-P Wald F statistic is above 10, indicating that the instrumental variable is valid [71]. Moreover, the core explanatory variable LFP positively affects population mortality, which is significant at the 1% level. As for the coefficient of *lnLFP*, the results indicate that, when LFP increases by 1%, the population mortality rate increases by 0.010167 unit. The instrumental variable model result compares with the basic results, and the basic results underestimate the negative impact of LFP on public health to a certain extent. Therefore, after a series of robustness tests, we conclude the robustness of the finding that LFP significantly positively affects mortality, thus negatively affecting public health.

### 4.3. Mechanism Analysis

This section utilizes Equations (2) and (3) to examine three mechanisms of the negative impact of LFP on public health. Table 4 shows the results of the checks of the three mechanism. Column 1 shows that LFP impacts public health expenditure negatively at the 1% significance level. This finding is consistent with most previous studies [66]. Column 2 shows that, once the variable *SPC* was added to the model, the coefficient of *lnLFP* (0.3624) is less than the coefficient in the basic model results, as shown in Table 2 (0.4933). The test results meet the three conditions proposed by Baron and Kenny (1986) [58]. The results indicate that LFP negatively impacts public health by reducing public health expenditure. The results also show that reductions in public health spending do increase population mortality and harm public health. Hence, Hypothesis 2 is verified. This partly provides new evidence regarding the uncertainty of the relationship between austerity and public health [24,25]. For the second mechanism, the coefficients in columns 3 and 4 indicate that LFP negatively impacts public health by hindering industrial upgrading. Hence, Hypothesis 3 is tested. Similarly, we can verify the third mechanism from the results in columns 5 and 6, which show the negative effect of LFP on public health via increased environmental pollution, providing sufficient evidence to establish Hypothesis 4. Moreover, we referred to Mackinnon et al. (1995) [72] and calculated that the shares of the three mediating effects is approximately 66.03%. This result shows that reducing public health expenditure, hindering industrial upgrading, and increasing environmental pollution are the three main channels through which LFP is significantly detrimental to public health.

### 4.4. Heterogeneity Analysis

**Figure 4:**
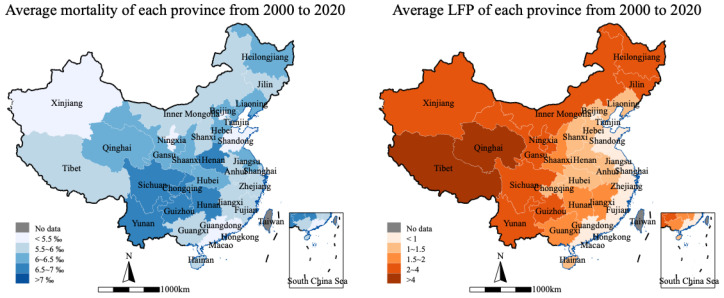
Average provincial differences from 2000 to 2020. (Source: *China Statistical Yearbook* (2001–2021) and *China Finance Yearbook* (2001–2021)).

Evidently, there is a large gap between China’s eastern and central-western regions. This regional gap is also evident in terms of public health and LFP. As can be seen in Figure 4, the darker the blue area, the higher the population mortality rate and the worse the public health level. Similarly, the darker the orange area, the worse the LFP. Is there a similar difference between the effects of LFP on public health? We classified the sample according to the eastern region versus other regions, and then conducted a heterogeneity analysis.

Table 5 shows the regression results of the heterogeneity analysis. The regression coefficient of the core explanatory variable is positive in column 1, but not significant; that is, LFP has no significant effect on public health in the eastern region. A possible reason for this is that the eastern region has better resource factor endowment and medical resources. Local governments can still meet public health expenditure expectations even in the face of LFP. To achieve economic growth under LFP, local governments in the eastern region can shift the industrial structure to high-tech industries rather than resource-intensive secondary industries. Therefore, the effect of LFP on public health is not significant in the eastern region.

Relative to the eastern region, the situation in Central and Western China is not optimistic. In column 2 of Table 5, the coefficient of the core explanatory variable is positively significant at the 1% level. This result indicates that in Central and Western China, LFP positively affects the population mortality rate (i.e., it is detrimental to public health). This result is consistent with the basic expectation that most provinces located in the central and western regions rely on resource-intensive industries for development [73]. In the face of fiscal pressure, local governments will further develop secondary industries, leading to markedly more severe environmental pollution. Severe environmental pollution coupled with poor healthcare infrastructure results in local governments curtailing public health expenditure under fiscal pressure, thereby severely undermining public health.

## 5. Conclusions and Policy Implications

### 5.1. Conclusions

Under the dual challenges of global downward economic pressure and the COVID-19 pandemic, the impact of LFP on public health needs to be studied. This paper theoretically and empirically analyzed the effect and impact mechanism of LFP on public health using two-way fixed-effects and mediating effects models with Chinese provincial panel data from 2000 to 2020. We found that LFP has a positive effect on population mortality, with the instrumental variable estimate results showing that when LFP increases by 1%, the population mortality rate will increase by 0.010167 per thousand people. This means that LFP has a significantly negative effect on public health. Moreover, the impacts are mainly felt through curtailing healthcare spending, hindering industrial upgrading, and increasing environmental pollution. The mediating effects of the three main channels account for 66.03% of the total effect. We conducted a series of robustness checks on the basic results by changing the model standard error cluster level, lagging the core explanatory variables by one period, and using the 2SLS method. The results of the robustness checks prove that our results are robust. Furthermore, we divided the sample into the eastern region and other regions for heterogeneity analysis. We found that the negative effect of LFP on public health is mainly found in Central and Western China, while the effect in Eastern China is not significant.

### 5.2. Policy Implications

This paper describes the behavioral responses of local governments in the context of LFP and the consequences for public health. It extends the research related to LFP. In addition, this paper presents some policy implications.

(1) Optimizing the fiscal system. The primary cause of LFP is the mismatch between financial and administrative powers. Accordingly, reducing the negative impact of fiscal pressure on public health requires starting from the central–local fiscal system. The tax revenue of local governments should be appropriately increased and the responsibility of local governments in terms of fiscal power and responsibility of affairs must be balanced. In addition, to optimize the tax-sharing system, the central government should appropriately decentralize fiscal power and increase the tax revenue of local governments, while increasing the right of local governments to self-management. Moreover, local governments should have sufficient funds to invest in public livelihood.
(2) Accelerating industrial upgrading. Local governments should increase their efforts to eliminate their dependence on resource-intensive industries, accelerate the upgrading of local industries to high-tech industries, and form an economic system and development model with innovation as the main leader. Industrial assistance from the eastern region to the central and western regions should be strengthened. Innovative green technologies should be introduced to reduce the impact of environmental pollution on public health.
(3) Improving the appraisal system of local officers. To alleviate local government officials’ excessive focus on GDP, it is necessary to evaluate these local officials from multiple perspectives, adjust the proportion of local economic development in the evaluation system, and increase the proportion of public livelihood facilities construction in the evaluation system. People’s subjective evaluation of public health facilities should be incorporated into the appraisal system.

This study completes the research on the effect and mechanism of LFP on public health, but still has some limitations. First, owing to a serious lack of data, this study did not use prefecture-level data with a larger sample size. Second, given that the COVID-19 pandemic affected all provinces in China in the same year, the impact of the pandemic is absorbed into the year fixed effect, making it impossible for us to study the behavior response of governments under the dual stresses of health emergency and fiscal pressure. In a follow-up study, we will attempt to discuss this research using cross-country data.

## Figures and Tables

**Table 1:** Descriptive statistics of the variables.

Classification	Variables	Obs.	Mean	Std. Dev.	Min	Max
Dependent Variable	*PublicHealth*	651	6.023	0.723	4.210	7.980
Independent Variables	*lnLFP*	651	0.349	0.865	−2.137	3.026
Mediator Variables	*SPC*	651	569.437	559.838	18.103	3944.535
	*ESI*	651	0.342	0.179	0.063	0.844
	*PM*	651	1.448	1.383	0.171	7.639
Control Variables	*GDPPC*	651	35.993	28.530	2.662	164.889
	*INDS*	651	1.099	0.354	0.189	2.023
	*RP*	651	4310.578	2775.093	258.000	12,624.000
	*URBAN*	651	51.518	15.777	19.328	89.600

Notes: Units of *PublicHealth*, *SPC*, *PM*, *GDPPC*, *RP* are ‰, CNY per capita, μg/m^3^ per 100 capita per year, CNY 1000 per capita and 10 thousands people, respectively. The units of *ESI*, *INDS* and *URBAN* are ratio. (Source: *China Statistical Yearbook* (2001–2021), *China Finance Yearbook* (2001–2021) and Atmospheric Composition Analysis Group).

**Table 2:** Basic results of the impact of local fiscal pressure on public health in China.

	Dependent Variable (Mortality)	
	(1)	(2)
lnLFP	0.4284 ***	0.4933 ***
	(0.0929)	(0.0860)
GDPPC		−0.0078 ***
		(0.0021)
INDS		−0.2712 ***
		(0.0949)
POP		0.0004 ***
		(0.0001)
URBAN		−0.0265 ***
		(0.0078)
Constant	5.8738 ***	6.1743 ***
	(0.0351)	(0.4892)
Observations	651	651
Province FE	YES	YES
Year FE	YES	YES
Adjusted R^2^	0.752	0.776
$\bar{\mathrm{CDw}}$	−0.98	−1.01
	(0.328)	(0.313)

Notes: *** *p* < 0.01. Robust standard errors in parentheses. To reduce the dependence of the weighted CD test statistic, $\bar{\mathrm{CDw}}$ is repeatedly performed with different weights by 100 times. Source: *China Statistical Yearbook* (2001–2021), *China Finance Yearbook* (2001–2021) and Atmospheric Composition Analysis Group.

**Table 3:** Robustness check results.

	Dependent Variable (Mortality)		
	(1)	(2)	(3)
	Cluster	L.lnLFP	2sls
lnLFP	0.4933 ***		1.0167 ***
	(0.1424)		(0.1786)
L.lnLFP		0.4352 ***	
		(0.0858)	
Constant	6.1743 ***	6.5581 ***	6.7135 ***
	(1.1999)	(0.4913)	(0.3496)
Observations	651	620	620
Control Variables	YES	YES	YES
Province FE	YES	YES	YES
Year FE	YES	YES	YES
R-squared	0.795	0.802	-
K-P Wald F	-	-	51.70
Province Cluster	31	-	-

Notes: In column 1, robust standard errors clustered at the province level are in parentheses. In column 2 and 3, robust standard errors in parentheses. K-P Wald F is Kleibergen-Paap rk Wald F statistic, which be used to check whether IV is weak. *** *p* < 0.01. (Source: *China Statistical Yearbook* (2001–2021), *China Finance Yearbook* (2001–2021) and Atmospheric Composition Analysis Group).

**Table 4:** Mechanism check results.

	First Mechanism		Second Mechanism		Third Mechanism	
	(1)	(2)	(3)	(4)	(5)	(6)
	SPC	Mortality	ESI	Mortality	PM	Mortality
lnLFP	−202.3652 ***	0.3624 ***	0.0259 **	0.4713 ***	0.3619 ***	0.4283 ***
	(51.1395)	(0.0903)	(0.0131)	(0.0873)	(0.0994)	(0.0857)
SPC		−0.0006 ***				
		(0.0001)				
ESI				0.8497 ***		
				(0.3144)		
PM						0.1798 ***
						(0.0651)
Constant	1638.6930 ***	7.2343 ***	−0.6707 ***	6.7442 ***	−0.4122	6.2484 ***
	(196.0541)	(0.5071)	(0.0667)	(0.5666)	(0.4366)	(0.4728)
Observations	651	651	651	651	651	651
Control Variables	YES	YES	YES	YES	YES	YES
Province FE	YES	YES	YES	YES	YES	YES
Year FE	YES	YES	YES	YES	YES	YES
Adjusted R^2^	0.917	0.797	0.936	0.798	0.961	0.800

Notes: Robust standard errors in parentheses. ** *p* < 0.05, *** *p* < 0.01. (Source: *China Statistical Yearbook* (2001–2021), *China Finance Yearbook* (2001–2021) and Atmospheric Composition Analysis Group).

**Table 5:** Heterogeneity analysis results.

	Dependent Variable (Mortality)	
	(1) Eastern Region	(2) Other Regions
LFP	0.1949	0.6144 ***
	(0.1197)	(0.1333)
Constant	4.2418 ***	8.8684 ***
	(0.8904)	(0.7879)
Observations	231	420
Control Variables	YES	YES
Province FE	YES	YES
Year FE	YES	YES
R-squared	0.839	0.803

Notes: The regional classification standard is based on *the China Statistical Yearbook*, the eastern region includes Beijing, Tianjin, Shandong, Jiangsu, Zhejiang, Fujian, Shanghai, Hainan, Hebei, Liaoning, Guangdong. Rest provinces belong to central or western regions. Robust standard errors in parentheses. *** *p* < 0.01. (Source: *China Statistical Yearbook* (2001–2021), *China Finance Yearbook* (2001–2021) and Atmospheric Composition Analysis Group).

## Data Availability

The data presented in this study are available upon reasonable request from the corresponding author.
